# CME ACTIVITY: Antimicrobial Drug Use and Resistance in Europe

**DOI:** 10.3201/eid1411.070616

**Published:** 2008-11

Medscape, LLC is pleased to provide online continuing medical education (CME) for this journal article, allowing clinicians the opportunity to earn CME credit. Medscape, LLC is accredited by the Accreditation Council for Continuing Medical Education (ACCME) to provide CME for physicians. Medscape, LLC designates this educational activity for a maximum of 0.75 *AMA PRA Category 1 Credits*™. Physicians should only claim credit commensurate with the extent of their participation in the activity. All other clinicians completing this activity will be issued a certificate of participation. To participate in this journal CME activity: (1) review the learning objectives and author disclosures; (2) study the education content; (3) take the post-test and/or complete the evaluation at **http://www.medscape.com/cme/eid**; (4) view/print certificate.

## Learning Objectives

Upon completion of this activity, participants will be able to:

- Identify the classes of antimicrobial drugs most commonly used in Europe.
- Describe patterns of antimicrobial drug use across regions in Europe.
- Identify the most widely used antimicrobial drugs by country in Europe.
- List European countries that show the highest antimicrobial drug resistance proportions.
- Describe the association between antimicrobial drug use and the emergence of resistance.

## EDITOR

**Anne Mather,** Technical Writer-Editor, *Emerging Infectious Diseases*. *Disclosure: Anne Mather has disclosed no relevant financial relationships.*

## CME AUTHOR

**Désirée Lie, MD, MSEd,** Clinical Professor, Family Medicine, University of California, Orange, California, USA; Director, Division of Faculty Development, UCI Medical Center, Orange. *Disclosure: Désirée Lie has disclosed no relevant financial relationships.*

## AUTHORS

Disclosures: **Nienke van de Sande-Bruinsma, MSc, PhD**; **Hajo Grundmann, MD, PhD**; **Didier Verloo, DVM**; **Edine Tiemersma, PhD**; **Jos Monen, MSc**; **Herman Goossens, MD, PhD**; and **Matus Ferech, MSc, PhD**, have disclosed no relevant financial relationships.

## Earning CME Credit

To obtain credit, you should first read the journal article. After reading the article, you should be able to answer the following, related, multiple-choice questions. To complete the questions and earn continuing medical education (CME) credit, please go to **http://www.medscape.com/cme/eid**. Credit cannot be obtained for tests completed on paper, although you may use the worksheet below to keep a record of your answers. You must be a registered user on Medscape.com. If you are not registered on Medscape.com, please click on the New Users: Free Registration link on the left hand side of the website to register. Only one answer is correct for each question. Once you successfully answer all post-test questions you will be able to view and/or print your certificate. For questions regarding the content of this activity, contact the accredited provider, CME@medscape.net. For technical assistance, contact CME@webmd.net. American Medical Association’s Physician’s Recognition Award (AMA PRA) credits are accepted in the US as evidence of participation in CME activities. For further information on this award, please refer to http://www.ama-assn.org/ama/pub/category/2922.html. The AMA has determined that physicians not licensed in the US who participate in this CME activity are eligible for *AMA PRA Category 1 Credits*™. Through agreements that the AMA has made with agencies in some countries, AMA PRA credit is acceptable as evidence of participation in CME activities. If you are not licensed in the US and want to obtain an AMA PRA CME credit, please complete the questions online, print the certificate and present it to your national medical association.

### Article Title: Antimicrobial Drug Use and Resistance in Europe

## CME Questions

1. Which of the following classes of antimicrobial drugs is least likely to be used in European countries?
  A. Lincosamides
  B. Fluoroquinolones
  C. Cotrimoxazole
  D. Macrolides
2. Which of the following regions in Europe has the highest outpatient utilization of antimicrobial drugs?
  A. Southern
  B. Northern
  C. Eastern
  D. Central
3. Which of the following is the most widely used antimicrobial drug class in Europe?
  A. Macrolides
  B. Penicillins
  C. Nonpenicillin beta-lactams
  D. Fluoroquinolones
4. Which of the following European countries showed both the greatest use of antimicrobial drugs in ambulatory care and the highest resistance proportions?
  A. France
  B. Croatia
  C. Italy
  D. Greece
5. Among European countries with high antimicrobial drug resistance rates, a robust and consistent association was most likely to be found between utilization and resistance for which of the following drugs?
  A. Penicillins and macrolides
  B. Lincosamides and macrolides
  C. Penicillins and streptogramins
  D. Penicillins and fluoroquinolones

### Activity Evaluation

**Table:** 

**1. The activity supported the learning objectives.**				
Strongly Disagree				Strongly Agree
1	2	3	4	5
**2. The material was organized clearly for learning to occur.**				
Strongly Disagree				Strongly Agree
1	2	3	4	5
**3. The content learned from this activity will impact my practice.**				
Strongly Disagree				Strongly Agree
1	2	3	4	5
**4. The activity was presented objectively and free of commercial bias.**				
Strongly Disagree				Strongly Agree
1	2	3	4	5

